# Does body composition mediate the association between moderate-to-vigorous physical activity and physical literacy in school-aged children? A cross-sectional mediation analysis

**DOI:** 10.3389/fpubh.2026.1772818

**Published:** 2026-06-23

**Authors:** Liu Long, Syed Ghufran Hadier, Syed Danish Hamdani, Syed Muhammad Zeeshan Haider Hamdani, Shaista Shireen Danish

**Affiliations:** 1 School of Physical Education, Suzhou University, Suzhou, Anhui, China; 2 Research Centre Wujia School, Suzhou University, Suzhou, Anhui, China; 3 College of Sports and Holistic Health, Sichuan Technology and Business University, Chengdu, China; 4 Department of Health Sciences and Sport, University of Stirling, Stirling, United Kingdom; 5 Stirling College, Chengdu University, Chengdu, Sichuan, China; 6 Department of Sports Sciences, Bahauddin Zakariya University, Multan, Pakistan; 7 Faculty of Sport Science, School of Exercise and Health, Shanghai University of Sport, Shanghai, China; 8 Division of Sports and Health, School of Sports Science, Department of Exercise Physiology, Beijing Sport University, Beijing, China

**Keywords:** CAPL-2, childhood obesity, mediation analysis, Pakistani school children, physical activity, physical literacy, South Punjab

## Abstract

**Background:**

Childhood obesity and physical inactivity are growing global public health concerns. Once predominantly observed in wealthier nations, these issues are now increasingly prevalent in low- and middle-income countries. We aimed to examine the relationship between moderate-to-vigorous physical activity (MVPA) and physical literacy (PL) among children aged 8–12 years, and to determine whether body mass index (BMI) mediates this association.

**Methods:**

We conducted a cross-sectional study of 1,360 children (8–12 years old, mean age 10.0 ± 1.4 years) from 85 higher secondary schools in South Punjab, Pakistan. Physical activity and PL were assessed using the Canadian Assessment of Physical Literacy (CAPL-2) protocol. Data analyses included descriptive statistics, independent *t*-tests, chi-square tests, Pearson correlations, and multivariate logistic regression. A regression-based mediation analysis was conducted to examine the mediating role of BMI in the MVPA–PL relationship. Age and sex were included as covariates. Statistical significance was set at *p* < 0.05 (two-tailed).

**Results:**

Overall, 79.9% of participants were of normal weight, 9.7% were overweight, and 5.3% were obese. Normal-weight children demonstrated significantly higher average weekly step counts, more MVPA days per week (5.04 ± 1.09 vs. 4.65 ± 1.17), and higher PL scores (53.76 ± 11.72 vs. 45.86 ± 9.87) than overweight or obese children (*p* < 0.001 for all comparisons). BMI was negatively associated with PA and PL measures (*r* = −0.136 to −0.300, *p* < 0.01), while MVPA and step count were positively associated with PL (*r* = 0.237 to 0.609, *p* < 0.01). In the mediation analysis, higher MVPA was associated with lower BMI (Path a: β = −0.377, SE = 0.078, *p* < 0.001), and lower BMI was associated with higher PL (Path b: β = −0.514, SE = 0.065, *p* < 0.001). MVPA also showed a strong direct association with PL (*c'* = 2.091, SE = 0.189, *p* < 0.001). The indirect effect of MVPA on PL through BMI was statistically significant [β = 0.194, 95% CI (0.107, 0.291)], accounting for approximately 8.5% of the total effect. Sex-stratified analyses indicated that the proportion of the MVPA–PL association mediated through BMI was slightly higher in girls (10.2%) than in boys (7.1%).

**Conclusion:**

This study provides the first evidence from a Pakistani pediatric population that BMI partially mediates the association between MVPA and physical literacy, though the majority of this relationship remained direct. Children with overweight and obesity demonstrated lower PA participation and reduced PL scores than normal-weight peers, while girls consistently underperformed boys despite comparable BMI and knowledge levels. These findings support the need for culturally relevant, gender-responsive, school-based programmes that promote physical activity while addressing weight-related barriers in under-resourced settings.

## 1 Introduction

Children's health and wellbeing are fundamental to the future of any nation. Childhood obesity and physical inactivity have emerged as critical challenges in global public health, and their consequences extend well beyond physical health to encompass cognitive, emotional, and social development (1). Childhood obesity, defined by excessive body fat accumulation relative to age- and sex-specific norms, is associated with increased risks of cardiovascular disease, type 2 diabetes, musculoskeletal disorders, and adverse psychosocial outcomes (2, 3). The World Health Organization has highlighted the scale of this concern: in 2016, more than 340 million children and adolescents worldwide were classified as overweight or obese (4). While these patterns were once more common in high-income countries, they are now increasingly evident in low- and middle-income settings, including South Asia, impairing not only physical health but also mental and emotional wellbeing (5).

Regular physical activity (PA) is widely recognized as central to addressing these challenges. It contributes to children's healthy growth and development, supporting motor skill acquisition, psychosocial functioning, cognitive development, and academic performance (6). Sustained engagement in PA, particularly moderate-to-vigorous physical activity (MVPA), is associated with improved mood, higher self-esteem, and reduced risk of chronic disease in youth (7). Importantly, active children tend to maintain healthier body weight, while excessive weight can, in turn, constrain physical participation and skill development suggesting a bidirectional relationship between activity and weight status (8).

Physical literacy (PL) is a multidimensional construct that encompasses the motivation, confidence, physical competence, knowledge, and understanding needed to engage in physical activity across the lifespan (9). The International Physical Literacy Association emphasizes that PL is not simply about movement skill, but about cultivating the desire and capacity to remain physically active throughout life (10). In this sense, PL may function both as an antecedent to PA, by supporting participation and engagement, and as an outcome of repeated movement experiences, through which children develop competence, confidence, and behavioral familiarity with PA over time (11, 12). The present study conceptualizes PL primarily as a developmental outcome associated with habitual PA and influenced by weight status. This perspective is supported by growing evidence showing that physically active children tend to achieve higher scores on validated PL assessments, including the CAPL-2 (13), as well as by developmental models proposing that regular participation in movement experiences is essential for PL development (14).

The association between weight status and PL is particularly relevant in this context. Children with higher BMI may face physical constraints such as reduced endurance, difficulty with agility and coordination tasks as well as psychosocial barriers such as lower confidence and reduced willingness to participate in PA (14). These factors can impede the development of physical competence and motivation, both of which are core dimensions of PL. BMI is therefore conceptualized in the present study as a mediator rather than a moderator of the MVPA–PL relationship. A moderating effect would suggest that the strength of the MVPA–PL association differs across BMI categories, whereas a mediating effect suggests that part of the association between MVPA and PL may operate through weight-related pathways. In practical terms, regular MVPA may be associated with healthier weight status, which may in turn support greater movement participation, competence, and confidence. This developmental pathway is biologically plausible and has received increasing empirical support in pediatric populations outside South Asia (13, 15).

Despite growing awareness of the obesity epidemic, the specific relationships among MVPA, weight status, and physical literacy remain underexplored in low- and middle-income settings. Pakistan has not been immune to rising childhood obesity trends (16). In South Punjab, obesity rates among school-aged children are estimated at 15%−20% above the national average, driven by urbanization, increasingly sedentary lifestyles, dietary change, and limited access to preventive health services (17). Studies in Lahore, Hyderabad, and Multan have reported overweight and obesity prevalence ranging from 15% to 24.5% in school-aged children (17–19), yet the relationship between activity levels, weight status, and PL in this population remains largely unexamined. No published study, to our knowledge, has investigated BMI as a mediator of the MVPA–PL relationship in Pakistani children.

To address these gaps, the present study examines the interrelationships among BMI, anthropometric adiposity indicators, physical activity metrics, and physical literacy scores in children aged 8–12 years from South Punjab, Pakistan. Specifically, we test whether BMI mediates the statistical association between MVPA and physical literacy. We also provide descriptive data on weight status, PA levels, and PL in this underrepresented population, and examine whether mediation pathways differ by sex. The findings are intended to inform locally relevant strategies for promoting physical activity and physical literacy, and to contribute to the broader evidence base on childhood health in South Asian contexts.

## 2 Methods

### 2.1 Study design and sampling

This study was conducted as part of the *Pakistan Initiative to Promote Physical Literacy* (PAK-IPPL) (9) and utilized a stratified random sampling approach targeting three administrative divisions of South Punjab: Multan, Bahawalpur, and Dera Ghazi Khan. These divisions were selected based on their demographic representativeness and geographical significance within the province (20, 21). The sample size was determined using Cochran's (1977) formula, $n=\frac{{\left(Z\right)}^{2}PQ}{e^{2}}\times D$ (22–24), where *Z* = 1.96 (corresponding to a 5% level of significance), *P* = 0.234 (anticipated proportion), *Q* = 0.766 (1-P), *e* = 0.002545 (desired precision level), and *D* = 5 (design effect). Based on these parameters, the required sample size was calculated as 1,359.8, rounded to 1,360 participants (25, 26).

A total of 87 higher secondary schools were initially selected using stratified random sampling, with 29 schools allocated per division. Two schools declined participation, yielding a final sample of 85 schools. From each participating school, a roster of students aged 8–12 years was obtained and 16 students per school were randomly invited to participate. An attrition rate of 11%, resulting from refusals or incomplete field testing, was addressed by recruiting additional participants from the same demographic group to preserve the target sample size. The final sample consisted of 455 students from Multan, 455 from Bahawalpur, and 450 from Dera Ghazi Khan, ensuring balanced regional representation.

### 2.2 Ethics approval

Ethical approval for this study was obtained from the Shanxi University School of Physical Education in 2020 (Approval Letter No: SXULL201912), in accordance with the Declaration of Helsinki. In addition to institutional approval, permission to conduct the study in schools was formally secured from the South Punjab Education Department at the provincial level and from individual school principals at the local level, ensuring dual oversight across administrative tiers. Participation was entirely voluntary. Written informed consent was obtained from the parents or legal guardians of all participating children prior to data collection, and children provided verbal assent after being briefed on the study's purpose and procedures in an age-appropriate manner and in their primary language.

### 2.3 Data collection procedure

**Figure 1:**
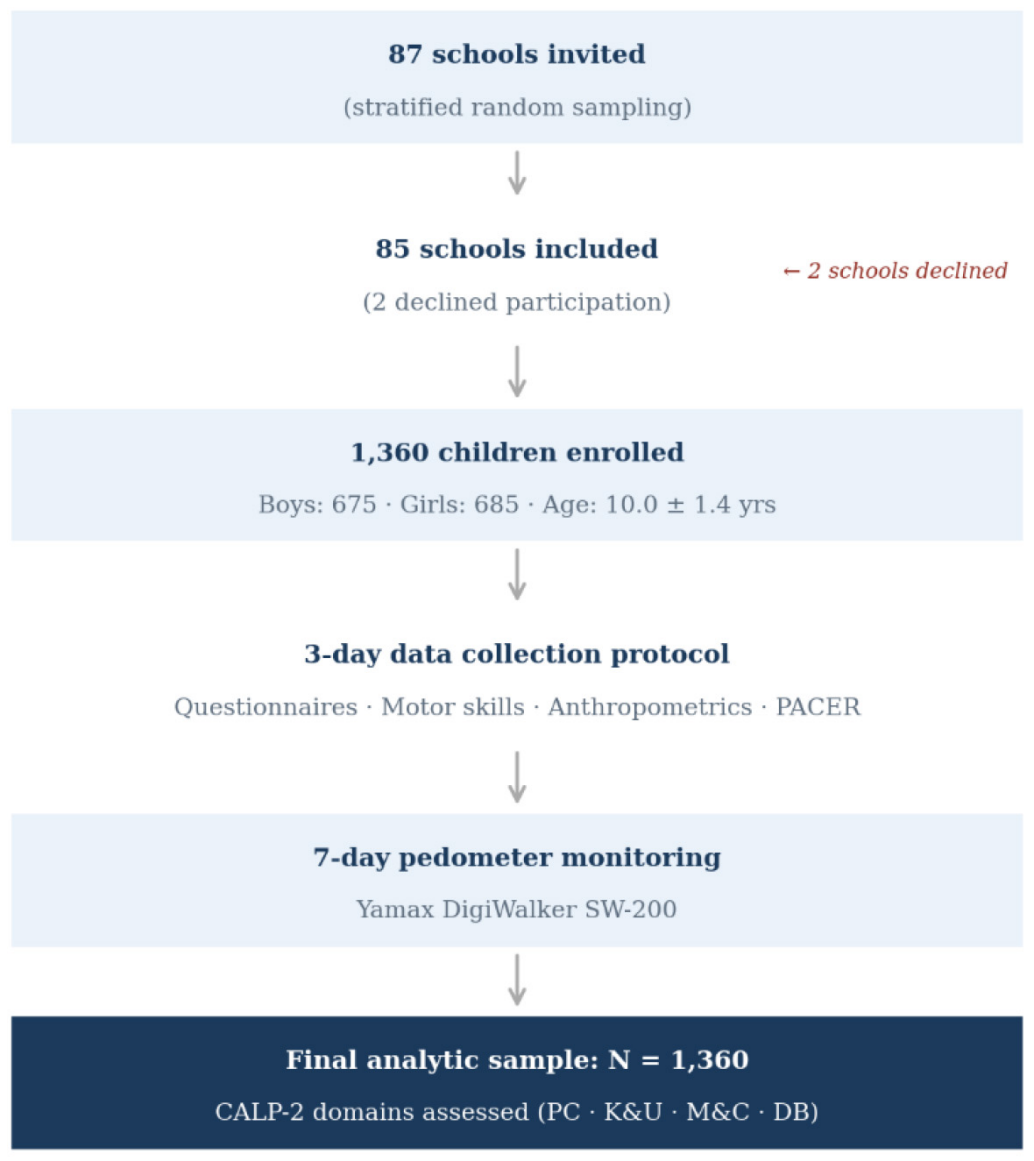
Flow chart of study procedure.

Data collection was carried out from September 12, 2020, to April 17, 2021, corresponding to the beginning of the academic year. Figure 1 provides a flowchart overview of the data collection process. For consistency and efficiency, we followed a standardized 3-day protocol at each school. Prior to visiting the schools, the research team coordinated with school administrators and sent information sheets to parents. Only students with documented parental consent and personal assent were enrolled.

On the first day, students were briefed on the study's purpose and procedures and completed self-reported demographic information and the CAPL-2 questionnaires. On the second day, students completed the Canadian Agility and Movement Skill Assessment (CAMSA) and the Plank Hold Test following standardized demonstrations. On the third day, anthropometric measurements were taken and the PACER test was administered. Pedometers were distributed at the end of the third day for seven consecutive days of step count monitoring.

Make-up sessions were arranged for students who were absent on any given day. Pedometer data management followed CAPL-2 manual guidelines (27): where 1 or 2 days of valid data were missing, the average of available days was used; where more than 2 days were missing, pedometer data for that child were considered invalid and excluded from step count analyses.

### 2.4 Measures

Demographics and Anthropometrics: Basic demographic information (including date of birth and sex) was obtained from school records and verified with students. Height was measured with the child standing barefoot and upright against a stadiometer, recorded to the nearest 0.1 cm. Weight was measured in light indoor clothing, with shoes and heavy items removed, using a calibrated digital scale, recorded to the nearest 0.1 kg.

Body mass index (BMI) was computed using the standard formula: $BMI=\frac{W\left(kg\right)}{Hm^{\left(2\right)}}$. Each child's BMI was then classified into weight status categories (underweight, healthy weight, overweight, or obese) according to age- and sex-specific percentile cut-offs from the US Centers for Disease Control and Prevention (CDC) growth charts (28). Underweight was defined as BMI < 5th percentile, healthy weight as 5th−84th percentile, overweight as 85th−94th percentile, and obese as ≥95th percentile for age and sex. Waist circumference (WC) and hip circumference (HC) were measured to assess body fat distribution. WC was measured with a non-stretch tape placed around the abdomen approximately 1 cm above the navel (umbilicus), ensuring the tape was horizontal and snug but not compressing the skin. HC was measured at the widest point of the buttocks. Both circumferences were recorded to the nearest 0.1 cm. The waist-to-hip ratio (WHR) was calculated as WC divided by HC (29), which provides an index of central adiposity (fat distribution).

### 2.5 Physical literacy

**Figure 2:**
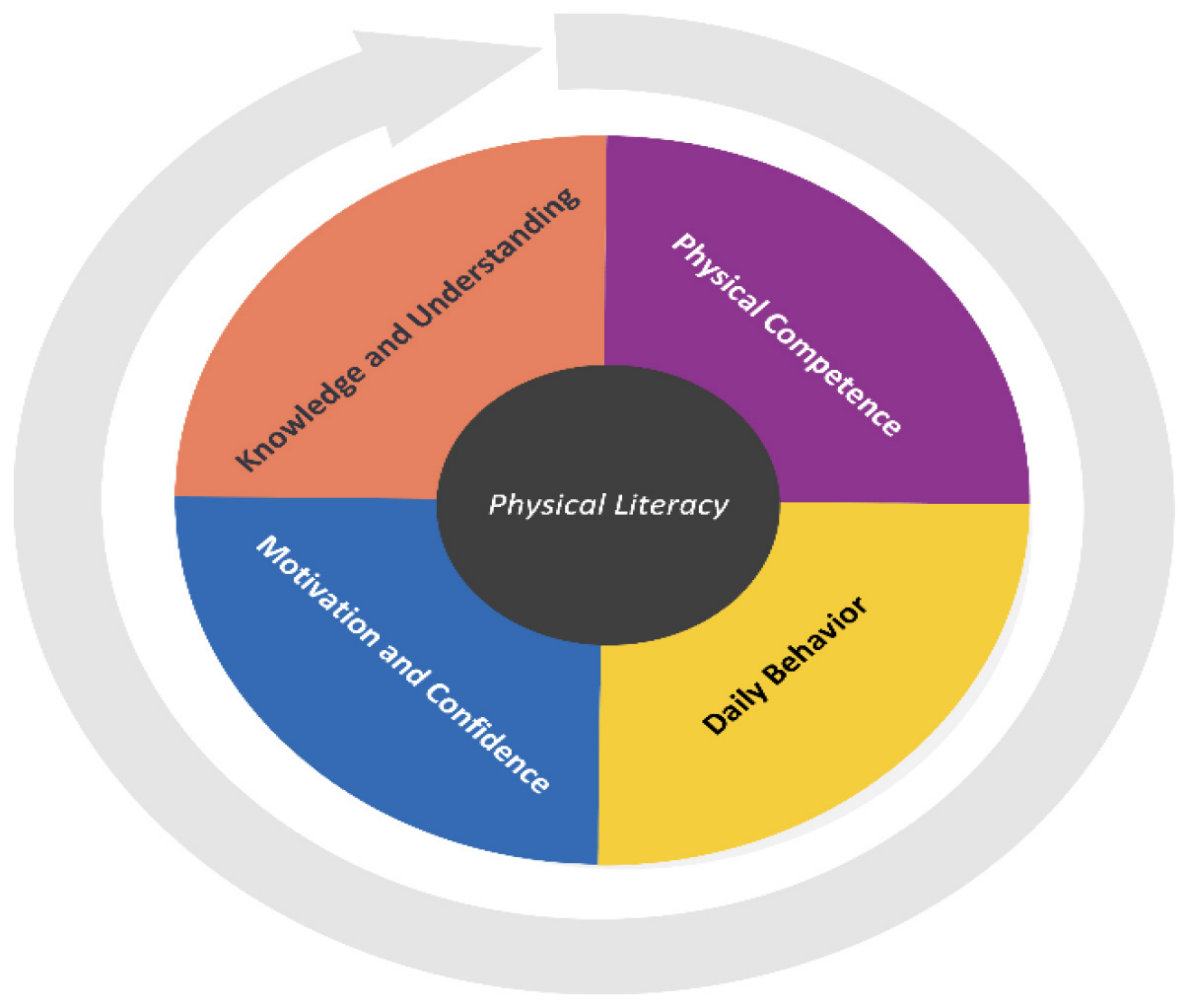
The core domains of physical literacy.

Physical literacy and physical activity levels were assessed using the CAPL-2 (27). CAPL-2 is a validated, multidimensional assessment framework comprising four domains (see Figure 2): Physical Competence (PC; 0–30 points), Knowledge and Understanding (K&U; 0–10 points), Motivation and Confidence (M&C; 0–30 points), and Daily Behavior (DB; 0–30 points), yielding a composite score out of 100. Domain-specific and composite scores permit both granular and overall assessment of children's physical literacy. Based on the composite score, participants are classified into four developmental levels: Beginning (below the 17th percentile), Progressing (17th−65th percentile), Achieving (65th−85th percentile), and Excelling (above the 85th percentile). CAPL-2 guidelines recommend that children attain at least the *Achieving* level to meet the standard for adequate physical literacy development (27). Prior to implementation, the CAPL-2 was linguistically adapted and cross-culturally validated for use with Pakistani children. The questionnaire components were translated into Urdu and validated in a pilot sample from South Punjab, confirming acceptable reliability and construct validity in this context (30). This represents the first implementation of the full CAPL-2 protocol in South Punjab, Pakistan.

Physical Competence (PC): The PC domain was evaluated using three standardized assessments, each contributing to a combined score of 30.

*Cardiovascular Endurance*: Aerobic capacity was assessed using the 20-meter Progressive Aerobic Cardiovascular Endurance Run (PACER), in which participants ran between two markers at incrementally increasing speeds. The total number of laps completed were converted into a score ranging from 1 to 10, based on CAPL-2 normative criteria (20).

*Muscular Strength*: Core and upper-body muscular strength were assessed using the Plank Hold Test. Children maintained a forearm plank position for as long as possible, with duration converted to a score of 1–10 (31, 32).

*Motor Skills*: Fundamental movement skills, including agility, jumping, and coordination, were assessed using the Canadian Agility and Movement Skill Assessment (CAMSA). Scores were assigned from 1 to 10 based on standardized scoring rubrics (33).

Knowledge and Understanding (K&U): This domain measured children's awareness of physical activity guidelines, health benefits, and strategies to improve physical competence. The assessment comprised four multiple-choice questions (one point each) and one fill-in-the-blanks activity (maximum six points), focusing on components related to endurance and skill development. The domain's total possible score was 10.

Motivation and Confidence (M&C): The M&C domain was assessed via a 12-item questionnaire spanning four sub-domains: Intrinsic Motivation, PA Self-Competence, Predilection, and Adequacy. Each item weighed 2.5 points, yielding a maximum domain score of 30. Collectively, the scores provide a comprehensive view of children's motivation, confidence, and readiness to engage in physical activity. Intrinsic Motivation: Assesses children's inherent interest and enjoyment in physical activity, reflecting their beliefs, attitudes, and emotional engagement. Physical Activity (PA) Self-Competence: Assesses perceived ability and confidence in performing physical activities, particularly in challenging contexts. Predilection: Assesses preference and inclination toward participating in diverse physical activities. Adequacy: Assesses children's perceived sufficiency in meeting the physical demands of activity and their self-assurance in their physical performance.

Daily Behavior (DB): DB domain evaluated children's physical activity engagement through both objective and self-reported data, with scores classified into the four CAPL-2 performance levels as Beginning, Progressing, Achieving, and Excelling based on the degree of adherence to recommended physical activity guidelines.

Physical Activity (Steps): Children's PA was objectively monitored using Yamax DigiWalker SW-200 pedometers (Yamax Corporation, Tokyo, Japan), selected for their demonstrated reliability (intraclass correlation coefficient > 0.90) and strong concordance with observer-verified step counts (34). Pedometers provide a practical and cost-effective means of quantifying ambulatory activity (35) and have shown consistent correlations with accelerometer data, oxygen consumption, and heart rate in prior research (36). Each participant wore the pedometer for seven consecutive days, with a valid weekly average calculated from complete days. Step count thresholds from CAPL-2 and prior empirical studies were applied to classify activity levels (37). Within the DB domain, pedometer-based step data contributed up to 25 of the 30 available points.

MVPA: Self-reported MVPA was obtained through a question in which children indicated the number of days in the preceding week on which they engaged in physical activity sufficient to noticeably elevate their heart rate, on a scale from 0 (no days) to 7 (every day). Self-reported MVPA contributed up to 5 of the 30 available DB domain points. It is noted that MVPA forms a scored component of the DB domain, which in turn contributes to the CAPL-2 composite score. To address the construct, overlap in mediation analyses, where MVPA serves as the independent variable, the primary mediation outcome was defined as a physical literacy composite score excluding the DB domain (PC + K&U + M&C; maximum = 70 points). The full CAPL-2 composite was retained as a secondary outcome for sensitivity analysis. The integration of both measurement approaches provides a more robust profile of individual physical activity patterns and supports targeted strategies for promoting active lifestyles among children.

### 2.6 Statistical analysis

All analyses were conducted using IBM SPSS Statistics Version 22. Descriptive statistics, including means, standard deviations, and frequency distributions, were computed for all key variables. Key statistical assumptions were verified prior to analysis: distributional properties were examined using the Shapiro–Wilk test, and outliers were identified using Z-scores with a threshold of ±5 SD; no observations exceeded this criterion. Independent-samples *t*-tests were used to examine sex-based differences in continuous variables, and chi-square tests were conducted to assess associations between categorical variables. Partial eta squared (ηp^2^) was reported as an effect size measure for group comparisons, interpreted as small (< 0.01), medium (< 0.06), or large (>0.14).

Pearson's correlation coefficients were calculated to examine associations among BMI, physical activity variables, and physical literacy domain scores. To avoid part-whole correlations arising from the structural overlap between step count, the Daily Behavior domain, and the CAPL-2 composite, the correlation matrix was computed using a construct-independent variable set that excluded the DB domain score and the full PL composite. These variables are reported separately for descriptive purposes.

Multivariate logistic regression was conducted to estimate odds ratios (ORs) with 95% confidence intervals for the likelihood of being overweight or obese, with normal-weight children as the reference group. Underweight children (*n* = 69) were excluded from this analysis. The DB domain score was excluded as a predictor due to severe collinearity with step count (VIF >90 when entered simultaneously); the final model included average weekly step count, self-reported MVPA days, physical literacy excluding DB, age, and sex, yielding an acceptable VIF range of 1.04–1.76.

**Figure 3:**
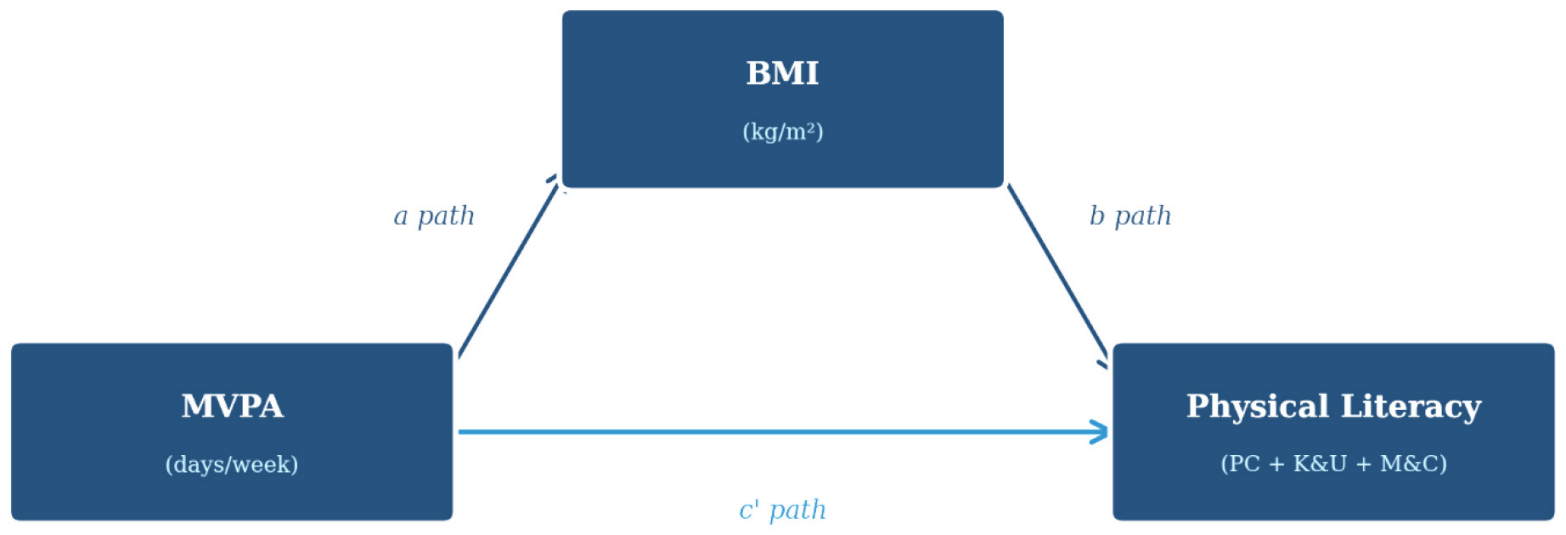
Conceptual mediation model: MVPA - BMI - Physical Literacy.

To examine the mediating role of BMI in the association between MVPA and PL, a regression-based mediation analysis was conducted using the PROCESS macro (Version 4.0, Model 4) for SPSS (Figure 3). This approach uses ordinary least squares regression to estimate the direct effect of MVPA on PL (path c'), the effect of MVPA on BMI (path a), the effect of BMI on PL controlling for MVPA (path b), and the indirect effect through BMI (path a × path b). BMI was treated as a continuous variable throughout. Self-reported MVPA days (0–7) served as the independent variable, and the primary outcome was the PL composite excluding the Daily Behavior domain (PC + K&U + M&C; maximum = 70 points). Age and sex were included as covariates in the full-sample model; sex was omitted from sex-stratified models. Bootstrap resampling with 5,000 iterations was applied to generate bias-corrected 95% confidence intervals for the indirect effect, which is considered statistically significant when the confidence interval excludes zero. Mediation analyses were conducted for the full sample and separately for boys and girls. A sensitivity analysis was conducted by re-running the mediation model using the full CAPL-2 composite (0–100, including DB) as the outcome to assess robustness of the primary findings. All tests were two-tailed with a significance threshold of *p* < 0.05.

## 3 Results

A total of 1,360 children (675 boys, 685 girls) participated in the study, with a mean age of 10.00 years (SD = 1.41, range 8–12). Table 1 summarizes the basic anthropometric and physical literacy characteristics by Sex. The overall mean height was 137.3 ± 11.2 cm, and mean body weight was 30.6 ± 8.2 kg, resulting in a mean BMI of 16.05 ± 3.1 kg/m^2^. Based on age- and sex-specific CDC percentile cut-offs, the majority of children (79.9%) were of normal weight, while 5.1% were classified as underweight, 9.7% as overweight, and 5.3% as obese. The distribution of weight status did not differ significantly between boys and girls (*p* = 0.348); however, a slightly higher proportion of girls fell in the underweight category (6.1% vs. 4.0%), while boys had a marginally higher prevalence of obesity (5.5% vs. 5.1%).

**Table 1:** Characteristics of the study participants by gender.

Characteristic	Boys (*n* = 675) x ±SD	Girls (*n* = 685) x ±SD	*p-value*
Age (years)	10.00 ± 1.42	10.00 ± 1.42	1.000
Height (cm)	137.79 ± 11.42	136.74 ± 11.25	0.088
Weight (kg)	30.82 ± 8.82	30.34 ± 8.41	0.308
BMI (kg/m^2^)	16.04 ± 3.19	16.06 ± 3.14	0.918
Avg weekly steps	8,115 ± 2,473	7,611 ± 1,814	< 0.001
MVPA (days/week)	5.17 ± 1.07	4.79 ± 1.09	< 0.001
DB Score (0–30)	12.85 ± 5.19	11.51 ± 3.87	< 0.001
PC Score (0–30)	17.39 ± 3.24	14.53 ± 4.02	< 0.001
PL Score (0–100)	55.10 ± 12.18	49.97 ± 10.75	< 0.001
PL excl. DB (0–70)	42.25 ± 8.01	38.46 ± 8.14	< 0.001
**Category**	**Boys**	**Girls**	***p-value***
Weight status distribution *n* (%)			
Underweight	27 (4.0%)	42 (6.1%)	0.348
Normal weight	546 (80.9%)	541 (79.0%)	0.348
Overweight	65 (9.6%)	67 (9.8%)	0.348
Obese	37 (5.5%)	35 (5.1%)	0.348

Data is presented as x: mean; SD, standard deviation (or percentages, as indicated); BMI, body mass index; MVPA, moderate-to-vigorous physical activity; DB, Daily Behavior domain; PL, Physical Literacy (CAPL-2); PL excl. DB, Physical Literacy excluding Daily Behavior domain (PC + K&U + M&C). Cohen's d: small = 0.20, medium = 0.50, large = 0.80. *p*-value significant at < 0.05.

Sex differences were observed across physical activity and physical literacy outcomes (Table 1). Boys recorded significantly higher average weekly step counts (8,115 ± 2,473 vs. 7,611 ± 1,814 steps, *p* < 0.001) and reported engaging in MVPA on more days per week (5.17 ± 1.07 vs. 4.79 ± 1.09 days, *p* < 0.001). Correspondingly, boys achieved higher DB domain scores (12.85 ± 5.19 vs. 11.51 ± 3.87 out of 30, *p* < 0.001) and higher PC domain scores (17.39 ± 3.24 vs. 14.53 ± 4.02 out of 30, *p* < 0.001). Boys also attained higher composite PL scores than girls (55.10 ± 12.18 vs. 49.97 ± 10.75 out of 100, *p* < 0.001), as well as higher PL scores excluding the DB domain (42.25 ± 8.01 vs. 38.46 ± 8.14 out of 70, *p* < 0.001).

Table 2 presents a comparison of anthropometric, physical activity, and physical literacy characteristics between normal-weight (NW; *n* = 1,087) and overweight or obese (OW/OB; *n* = 204) children, along with multivariate logistic regression results. Compared with normal-weight children, the OW/OB group had significantly higher body weight, BMI, waist circumference, and hip circumference (all *p* < 0.001), and a marginally higher waist-to-height ratio (η*p*^2^ = 0.006). Normal-weight children recorded significantly higher average weekly step counts (8,149 ± 2,146 vs. 6,294 ± 1,734; η*p*^2^ = 0.095), more MVPA days per week (5.04 ± 1.09 vs. 4.65 ± 1.17), and higher PL scores both excluding DB (41.04 ± 8.22 vs. 36.65 ± 7.81) and on the full composite (53.76 ± 11.72 vs. 45.86 ± 9.87; all *p* < 0.001).

**Table 2:** Multivariate logistic regression comparing normal weight vs. overweight/obese children.

Characteristic	NW (*n* = 1,087) x ±SD	OW/OB (*n* = 204) x ±SD	*p-value*	*ηp^2^*	OR [95% CI]
**Gender**	546 boys (50.3%) 541 girls (49.7%)	102 boys (49.5%) 102 girls (50.5%)	1.000	—	0.897 [0.614–1.310]
Height (cm)	136.33 ± 11.23	140.88 ± 10.94	<0.001	0.022	—
Weight (kg)	28.88 ± 6.75	42.78 ± 7.44	<0.001	—	—
BMI (kg/m^2^)	15.38 ± 2.04	21.44 ± 1.65	<0.001	—	—
WC (cm)	58.33 ± 8.19	65.46 ± 9.11	<0.001	0.089	—
HC (cm)	64.42 ± 7.62	70.30 ± 9.25	<0.001	0.069	0.772 (0.692–0.861)
Avg weekly steps (per 1,000)	8,149 ± 2,146	6,294 ± 1,734	<0.001	0.095	0.22 [0.17–0.28]
MVPA (days/week)	5.04 ± 1.09	4.65 ± 1.17	<0.001	0.016	1.001 [0.834–1.201]
PL excl. DB (0–70)	41.04 ± 8.22	36.65 ± 7.81	<0.001	0.037	0.973 [0.945–1.001]
DB Score (0–30)	12.72 ± 4.64	9.21 ± 3.41	<0.001	0.076	—
PL Score (0–100)	53.76 ± 11.72	45.86 ± 9.87	<0.001	0.060	—
Model fit statistics					
Nagelkerke *R^2^*	0.310	—	—	—	—
Model *χ^2^ p-value*	<0.001	—	—	—	—
VIF range (model vars)	1.04–1.76 (acceptable)	—	—	—	—

Data is presented as x mean; SD, standard deviation, % percentage; NW, Normal weight; OW, Overweight; BMI, body mass index; WC, waist circumference; HC, hip circumference; MVPA, moderate to vigorous physical activity; DB, Daily behavior; PL, Physical Literacy; OR, Odds Ratio; CI, Confidence Interval; ηp^2^, partial eta squared (small <0.01, medium <0.06, large > 0.14). PL excl. DB, Physical Literacy excluding Daily Behavior domain (PC + K&U + M&C). Odds ratio for average weekly steps is reported per 1,000 steps to improve interpretability. Steps and MVPA entered as activity predictors; DB excluded to resolve multicollinearity.

In the multivariate logistic regression (Nagelkerke *R*^2^ = 0.310, χ^2^ *p* < 0.001; VIF range 1.04–1.76), higher weekly step count was the strongest independent predictor of normal weight status [OR = 0.22 per 1,000 steps, 95% CI (0.17–0.28), *p* < 0.001]. Self-reported MVPA days were not independently significant after controlling for step count, age, and sex [OR = 1.001, 95% CI (0.834–1.201), *p* > 0.05], indicating that the protective association of physical activity with weight status is more reliably captured by objective step count in this sample.

**Figure 4:**
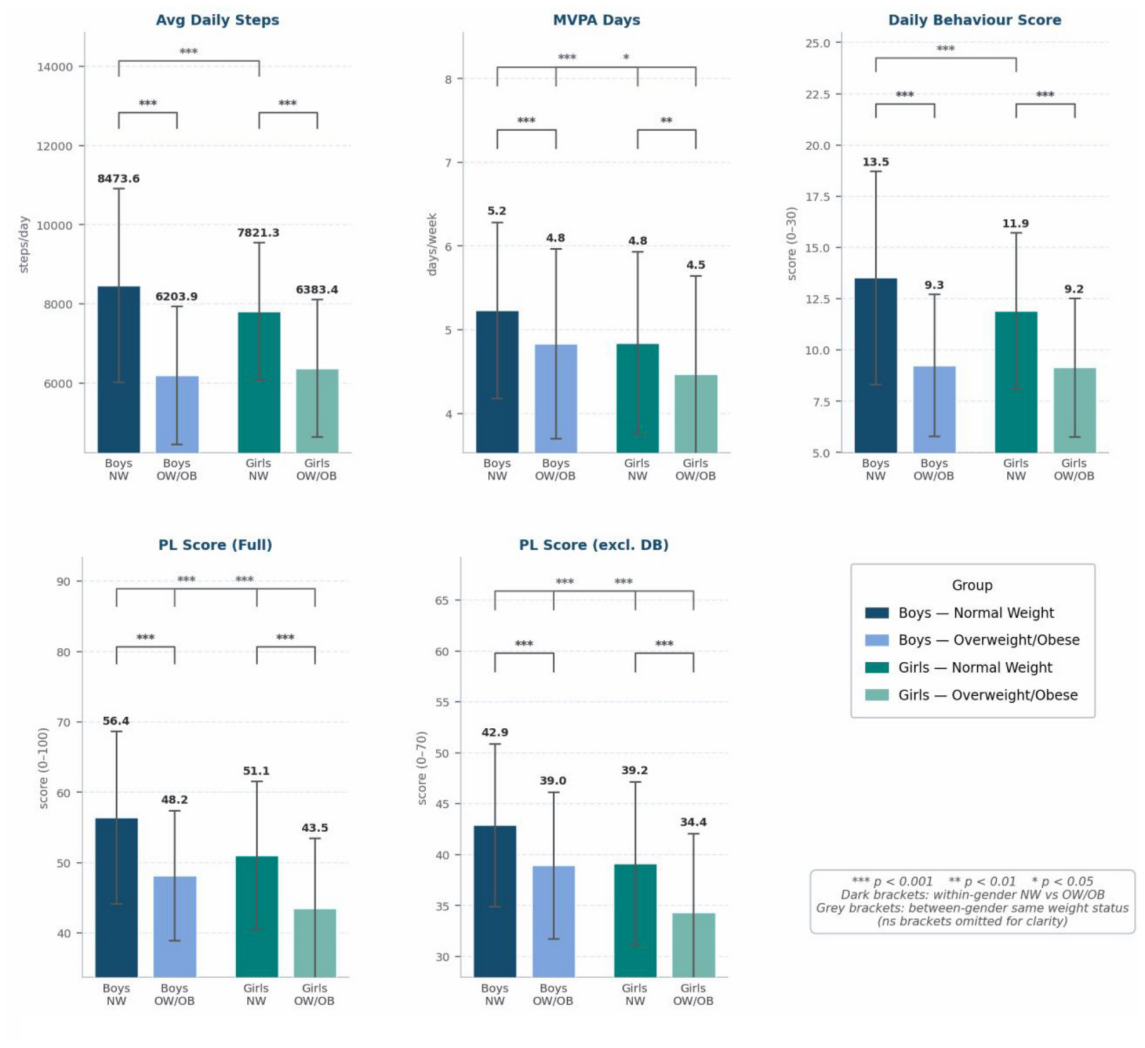
Physical activity and physical literacy by gender and weight status (Mean ± SD; error bars = ±1 SD; NW = Normal Weight, OW/OB = Overweight/Obese).

Figure 4 illustrates mean physical activity and physical literacy scores by sex and weight status group, with error bars representing ±1 SD. Across all PA and PL indicators, normal-weight children consistently outperformed their overweight or obese peers, regardless of sex. Among boys, those of normal weight recorded higher average step counts, more MVPA days, higher DB domain scores, and higher composite PL scores compared with overweight or obese boys. The same pattern was evident among girls, with normal-weight girls achieving substantially higher PA and PL scores than their OW/OB counterparts.

Table 3 presents the distribution of participants across CAPL-2 interpretive categories for daily step count, MVPA, DB score, and composite PL. For daily step count, the majority of children fell in the Progressing category (47.9%), with only 14.9% reaching Excelling. A similar pattern was observed for self-reported MVPA, with 47.7% classified as Progressing and 14.7% as Excelling. The DB domain showed a less favorable distribution, with 56.4% of children classified as Beginning. For composite physical literacy, 55.7% of the sample was in the Progressing category, and only 12.4% reached the Excelling level, while 15.9% remained at Beginning.

**Table 3:** Status of children PA, MVPA, DB, and PL according to CAPL-2 interpretation categories.

Variable	Interpretive categories	Total (1,360) *N* (%)
**PA behavior** (Daily steps)	Beginning (< 7,169)	233 (17.1)
	Progressing (7,170–7,367)	651 (47.9)
	Achieving (7,368–7,635)	273 (20.1)
	Excelling (>7635)	203 (14.9)
**MVPA** (Days)	Beginning (< 4)	235 (17.3)
	Progressing (4)	649 (47.7)
	Achieving (5–6)	276 (20.3)
	Excelling (>6)	200 (14.7)
**Daily behavior score** (0–30)	Beginning (< 10.05)	767 (56.4)
	Progressing (10.05 to 12.00)	332 (24.4)
	Achieving (12.00 to 13.86)	129 (9.5)
	Excelling (>13.86)	131 (9.7)
**Physical literacy** (0–100)	Beginning (< 41.99)	216 (15.9)
	Progressing (42.00 to 55.00)	758 (55.7)
	Achieving (55.01 to 62.00)	217 (16.0)
	Excelling (>62.00)	169 (12.4)

PA Behavior: measured in the number of average weekly steps; Self-reported MVPA: measured in # of self-reported days.

**Figure 5:**
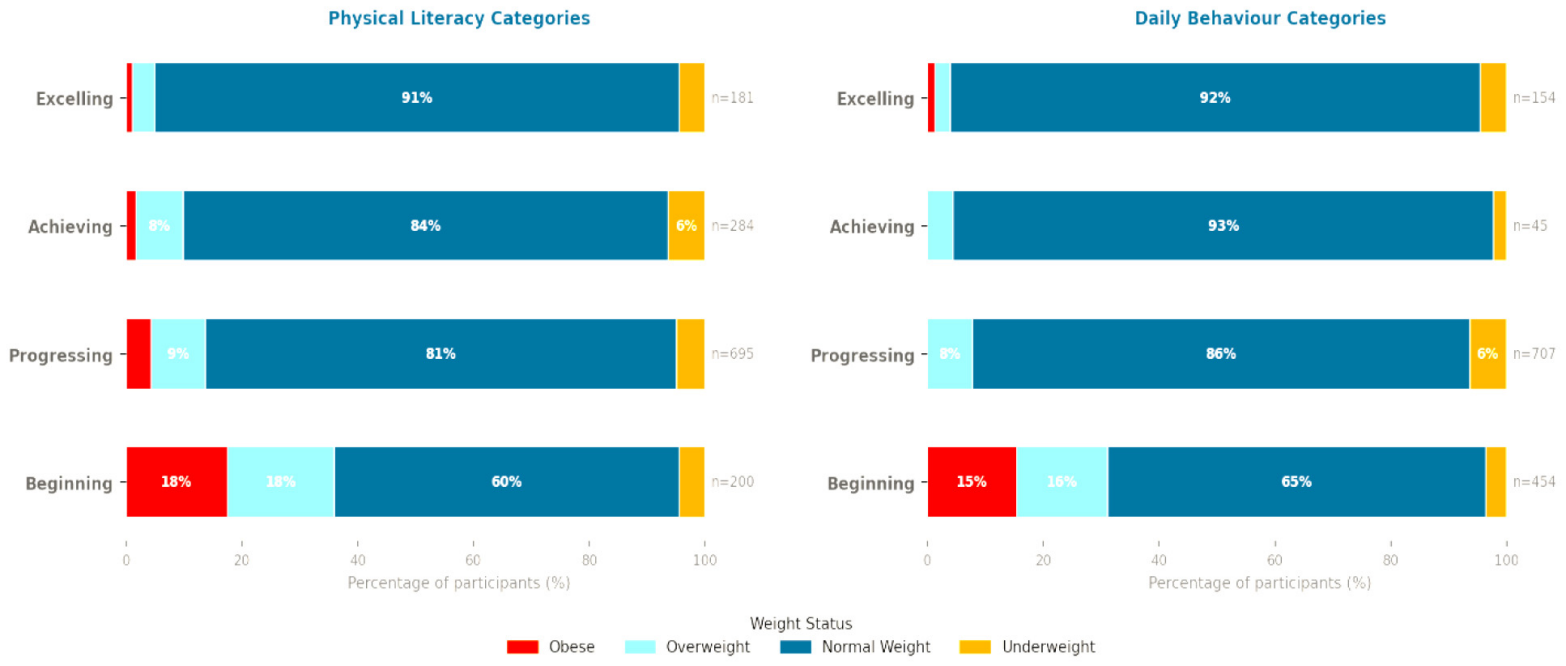
Weight status distribution across CAPL-2 Interpretive Categories (100% stacked bars; proportions within each category).

Figure 5 illustrates the distribution of weight status categories across CAPL-2 interpretive levels for PL, daily behavior, MVPA, and step count. Across all four measures, normal-weight children constituted the largest proportion within each interpretive category, and this dominance became increasingly pronounced at higher performance levels. Among children classified as Achieving or Excelling in PL and DB, the great majority were normal weight, with overweight and obese children representing a notably smaller share. Conversely, the Beginning category contained a higher relative proportion of overweight and obese children across all measures. Underweight children were distributed across all categories without a discernible pattern, suggesting that low body weight does not confer a systematic advantage in physical activity or physical literacy outcomes in this sample. The Progressing category, which contained the largest number of participants across all measures, reflected a broadly representative distribution of weight status, indicating that children of varying adiposity levels are still developing adequate physical activity habits and PL. Notably, the Excelling category was the smallest across all measures, with only 12.4% of children reaching this level in PL.

**Figure 6:**
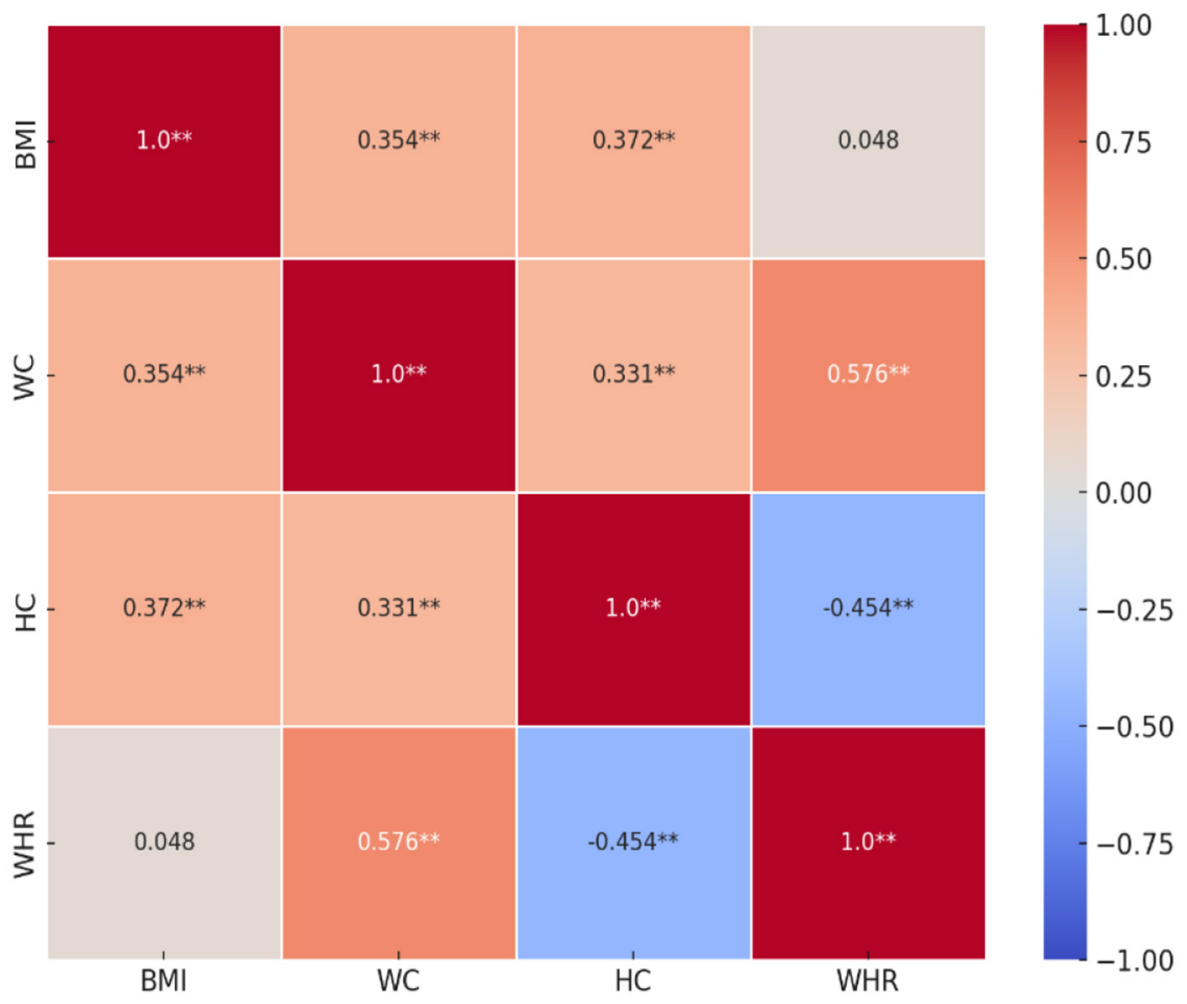
Correlation heatmap of BMI and anthropometric indicators of adiposity in children aged. WC, waist circumference; HC, hip circumference; WHR, waist-hip ratio; **Correlation is significant at the 0.01 level (2-tailed).

Anthropometry correlations: Figure 6 presents the correlation heatmap for anthropometric and adiposity indicators. BMI was significantly and positively correlated with both waist circumference (*r* = 0.354, *p* < 0.01) and hip circumference (*r* = 0.372, *p* < 0.01), consistent with the expectation that children with higher overall adiposity tend to have larger abdominal and hip girths. Waist and hip circumferences were also positively correlated with each other (*r* = 0.331, *p* < 0.01). The WHR displayed a distinct pattern: it was moderately correlated with waist circumference (*r* = 0.576, *p* < 0.01) but negatively correlated with hip circumference (*r* = −0.454, *p* < 0.01), indicating that WHR captures fat distribution rather than overall adiposity. Notably, WHR showed a negligible and non-significant correlation with BMI (*r* = 0.048, *p* > 0.05).

**Figure 7:**
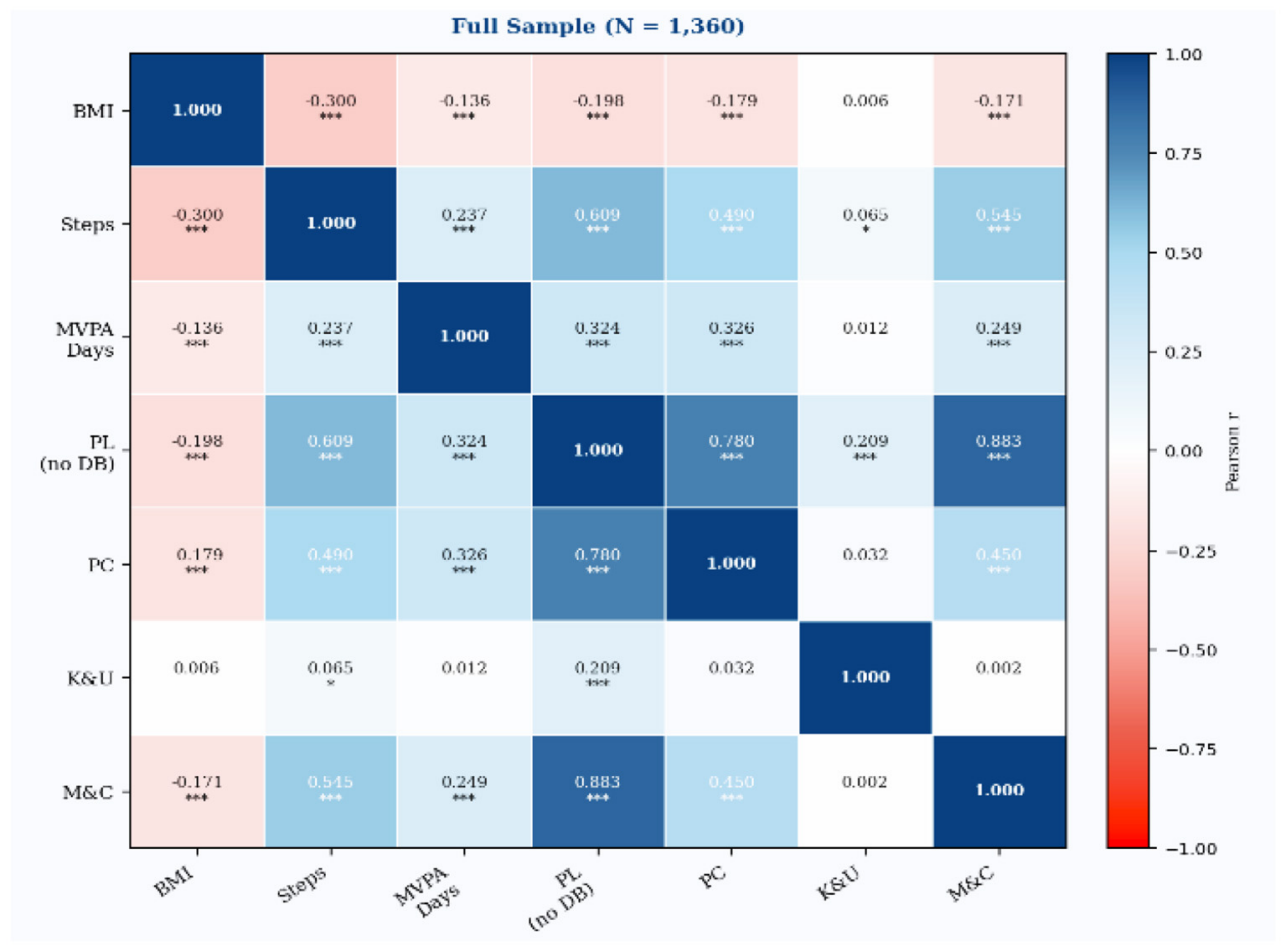
Correlation heatmap: BMI, physical activity, and physical literacy. PL (no DB) = Physical Literacy excluding Daily Behavior domain (PC + K&U + M&C, max = 70). Steps = average weekly step count; MVPA = self-reported days/week. ***p < 0.001, *p < 0.05 (two-tailed, *N* = 1,360).

Physical activity and PL correlations: Figure 7 presents the correlation heatmap using a construct-independent variable set that excludes the DB domain score and the full PL composite, to avoid part-whole correlations arising from the structural overlap between these measures and the predictor variables. BMI was negatively associated with average weekly step count (*r* = −0.300, *p* < 0.001), self-reported MVPA days (*r* = −0.136, *p* < 0.001), PL excluding DB (*r* = −0.198, *p* < 0.001), PC (r = −0.179, p < 0.001), and M&C (*r* = −0.171, *p* < 0.001). Notably, BMI showed no significant association with K&U (*r* = 0.006, *p* = 0.820), indicating that weight status is unrelated to children's cognitive knowledge of physical activity in this sample. Among PA and PL variables, step count was positively correlated with PL excluding DB (*r* = 0.609, *p* < 0.001), PC (*r* = 0.490, *p* < 0.001), and M&C (*r* = 0.545, *p* < 0.001). Self-reported MVPA days showed moderate positive correlations with PL excluding DB (*r* = 0.324, *p* < 0.001), PC (*r* = 0.326, *p* < 0.001), and M&C (*r* = 0.249, *p* < 0.001), but no significant association with K&U (*r* = 0.012, *p* = 0.650), consistent with the pattern observed for BMI.

### 3.1 Mediation analysis

**Figure 8:**
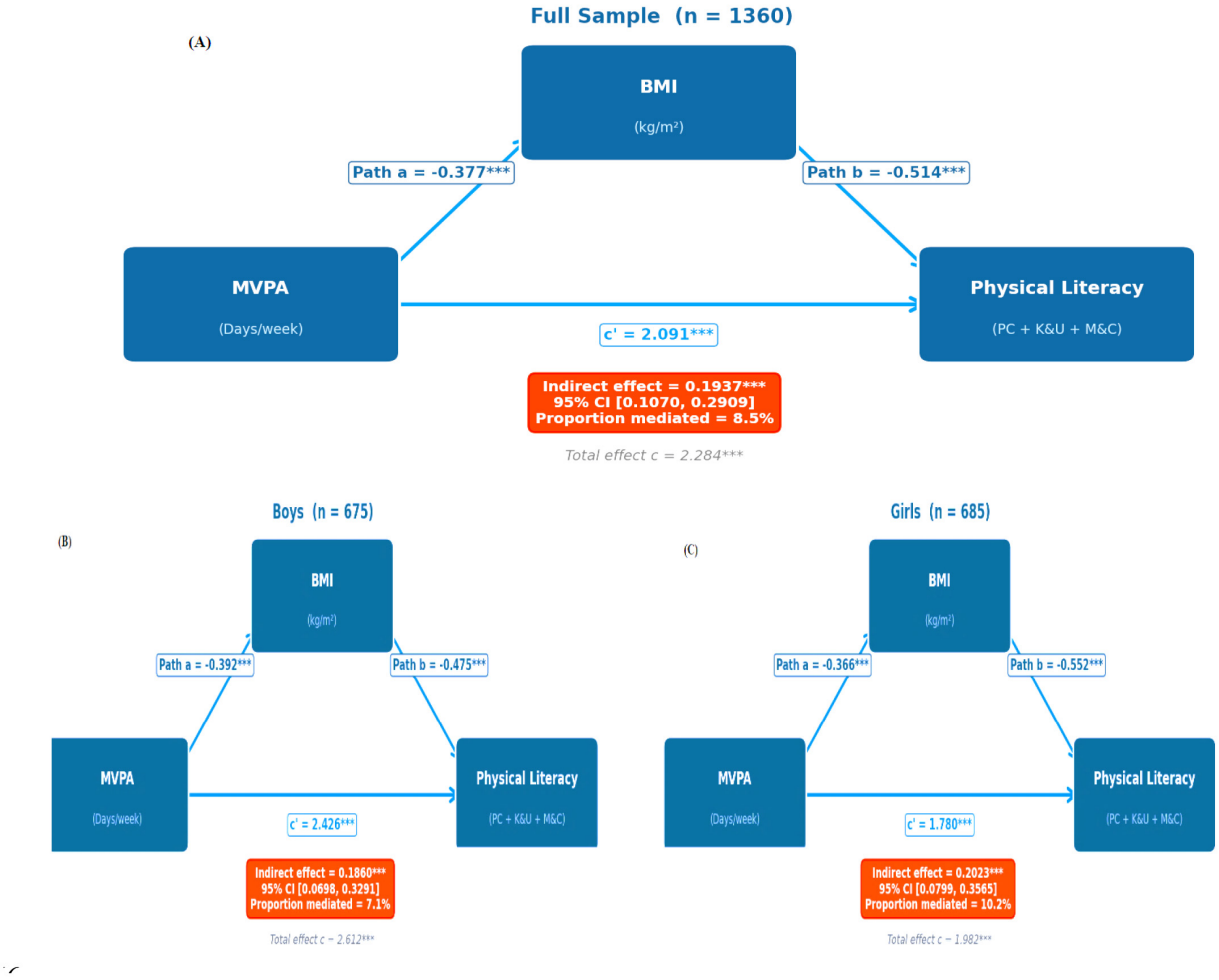
**(A)** MVPA - BMI - Physical Literacy (excluding daily behavior); **(B)** Boys stratified mediation: MVPA - BMI - Physical Literacy (excluding Daily Behavior); **(C)** Girls Stratified Mediation: MVPA - BMI - Physical Literacy (excluding Daily Behavior). ***p < 0.001.

The results of the regression-based mediation analysis are presented in Table 4 and Figure 8, for the full sample and separately for boys and girls. All models used self-reported MVPA days as the independent variable, continuous BMI as the mediator, and the physical literacy composite excluding the DB domain (PC + K&U + M&C; maximum = 70 points) as the outcome, with age and sex included as covariates in the full-sample model.

**Table 4:** Test of mediation using a bootstrap analysis with a 95% confidence interval.

Effect	Pathway	β (unstd.)	SE	*t*	95% CI	*p*
Model 1: Full sample (*n* = 1,360) — Covariates: Age, Sex						
Path a	MVPA → BMI	−0.3768	0.0777	−4.849	[−0.529, −0.225]	< 0.001
Path b	BMI → PL (excl. DB)	−0.5141	0.0653	−7.870	[−0.642, −0.386]	< 0.001
Direct c'	MVPA → PL (excl. DB)	2.0908	0.1885	11.091	[1.721, 2.461]	< 0.001
**Indirect** ^**†**^	MVPA → BMI → PL	0.1937	0.0476	—	[0.107, 0.291]	< 0.001
Total c	MVPA → PL	2.2845	—	11.957	[1.916, 2.653]	< 0.001
**Proportion mediated**	—	8.5%	—	—	—	—
Model 2: Boys only (*n* = 675) — Covariate: Age						
Path a	MVPA → BMI	−0.3916	0.1131	−3.463	[−0.614, −0.170]	< 0.001
Path b	BMI → PL (excl. DB)	−0.4750	0.0896	−5.299	[−0.651, −0.299]	< 0.001
Direct c'	MVPA → PL (excl. DB)	2.4260	0.2651	9.153	[1.905, 2.947]	< 0.001
**Indirect** ^**†**^	MVPA → BMI → PL	0.1860	0.0660	—	[0.070, 0.329]	< 0.001
Total c	MVPA → PL	2.6120	—	9.747	[2.082, 3.142]	< 0.001
**Proportion mediated**	—	7.1%	—	—	—	—
Model 3: Girls only (*n* = 685) — Covariate: Age						
Path a	MVPA → BMI	−0.3664	0.1070	−3.426	[−0.576, −0.157]	< 0.001
Path b	BMI → PL (excl. DB)	−0.5522	0.0952	−5.803	[−0.739, −0.365]	< 0.001
Direct c'	MVPA → PL (excl. DB)	1.7800	0.2681	6.640	[1.254, 2.306]	< 0.001
**Indirect** ^**†**^	MVPA → BMI → PL	0.2023	0.0714	—	[0.080, 0.357]	< 0.001
Total c	MVPA → PL	1.9823	—	7.285	[1.454, 2.510]	< 0.001
**Proportion mediated**	—	10.2%	—	—	—	—

β = unstandardised regression coefficient; SE, standard error; t, t-statistic (for OLS paths); CI, confidence interval. ^†^Indirect effect SE and CI derived from 5,000 bootstrap resamples with replacement; t-statistic not applicable for indirect effect. MVPA = self-reported days/week (0–7); BMI = continuous (kg/m^2^). PL excl. DB = PC + K&U + M&C (max 70). Proportion mediated = indirect effect / total effect × 100. All models controlled for age; combined model additionally controlled for sex.

In the full-sample model (Model 1; *n* = 1,360), MVPA was significantly and negatively associated with BMI (Path a: β = −0.377, SE = 0.078, *p* < 0.001), indicating that children who reported more days of MVPA tended to have lower BMI values, after controlling for age and sex. BMI was in turn significantly and negatively associated with PL (Path b: β = −0.514, SE = 0.065, *p* < 0.001), such that higher BMI was associated with lower PL scores, independent of MVPA. The direct association between MVPA and PL, after accounting for the mediating role of BMI, remained strong and positive (*c'* = 2.091, SE = 0.189, *p* < 0.001), indicating that MVPA is associated with higher PL through pathways beyond weight status alone. The indirect effect of MVPA on PL through BMI was statistically significant [β = 0.194, SE = 0.048, 95% bootstrap CI (0.107, 0.291)], representing approximately 8.5% of the total effect (total *c* = 2.285, *p* < 0.001). The positive sign of the indirect effect reflects the product of two negative paths higher MVPA is associated with lower BMI, and lower BMI is associated with higher PL yielding a net positive indirect contribution of MVPA to PL through this pathway. These results indicate partial mediation, whereby BMI accounts for a modest but statistically significant portion of the association between MVPA and PL.

In the sex-stratified models, the mediation pattern was consistent in direction across both boys and girls, though with notable differences in magnitude. Among boys (Model 2; *n* = 675), Path a (β = −0.392, SE = 0.113, *p* < 0.001) and Path b (β = −0.475, SE = 0.090, *p* < 0.001) were both significant, and the indirect effect was statistically significant [β = 0.186, 95% CI (0.070, 0.329)], accounting for 7.1% of the total effect (total *c* = 2.612, *p* < 0.001). Among girls (Model 3; *n* = 685), Path b was somewhat stronger than in boys (β = −0.552, SE = 0.095, *p* < 0.001), and the indirect effect was likewise significant [β = 0.202, 95% CI (0.080, 0.357)], representing a slightly higher proportion of the total effect at 10.2% (total *c* = 1.982, *p* < 0.001). This suggests that the BMI-mediated pathway from MVPA to physical literacy may be of somewhat greater relative importance for girls than for boys in this sample, consistent with the stronger negative association between BMI and PL observed among girls.

To assess the robustness of these findings to the definition of the PL outcome, a sensitivity analysis was conducted using the full CAPL-2 composite score (0–100, including the DB domain) in place of the DB-excluded composite. The indirect effect remained statistically significant [β = 0.335, 95% CI (0.192, 0.491)], and the proportion of the total effect mediated through BMI was virtually unchanged at 8.3%. The convergence of results across both PL operationalization confirms that the mediation finding is not an artifact of construct overlap between the MVPA predictor and the DB component of the PL outcome.

## 4 Discussion

This study examined the relationships between physical activity, body weight, and physical literacy in 1,360 school-aged children from South Punjab, Pakistan, and specifically tested whether BMI mediates the statistical association between MVPA and PL. Three findings stand out. First, children classified as overweight or obese were considerably less active and had markedly lower PL scores than their normal-weight peers, and this gap was apparent across both objective and self-reported measures of activity. Second, and most importantly, while BMI was found to partially mediate the MVPA–PL association, most of that relationship was direct, operating through the developmental benefits of physical activity itself rather than through weight-related mechanisms. Third, boys consistently outperformed girls across every activity and PL measure assessed, despite being physically indistinguishable in terms of body size, pointing to sociocultural rather than biological explanations for the gap. To our knowledge, this is the first study in Pakistan to formally test and quantify the BMI-mediated pathway from MVPA to PL, and the findings offer both a useful baseline for this underrepresented population and a set of practically actionable insights for those working to improve children's health and physical literacy in South Asian school settings.

### 4.1 Association between MVPA and physical literacy

Normal-weight children demonstrated higher daily step counts, more MVPA days, and higher PL scores than their overweight and obese peers, a pattern consistent with findings from Canadian, Spanish, and Chinese samples (38, 39), and with a companion study from the same dataset in which cardiorespiratory fitness emerged as a partial mediator of the MVPA–PL relationship (3). The domain-specific pattern adds interpretive depth. BMI was negatively associated with physical competence (r = −0.179) and motivation and confidence (r = −0.171), but showed no meaningful association with knowledge and understanding (r = 0.006). This dissociation indicates that excess weight constrains the physical and affective domains of PL, through reduced ease of movement, lower perceived competence, and restricted physical participation but does not impede the cognitive domain. For practitioners, this implies that intervention programmes targeting overweight children should prioritize building PC and M&C rather than delivering knowledge content, which appears relatively preserved in this group (11).

The logistic regression model identified average weekly step count but not self-reported MVPA as a significant independent predictor of weight status after accounting for age, sex, and PL. This finding is consistent with established evidence on the measurement limitations of self-reported PA in children, including recall bias, social desirability effects, and difficulty distinguishing intensity levels (40). Step count as measured by pedometry captured habitual ambulatory activity in a way that self-report did not, reinforcing the case for objective monitoring where feasible in population surveillance. On adiposity measurement, the near-zero correlation between WHR and BMI (r = 0.048) in this sample confirms that these two indices are not interchangeable and capture distinct aspects of body composition, overall adiposity in the case of BMI, and fat distribution in the case of WHR (41). For resource-constrained settings, BMI and waist circumference together offer the most practical combination for population monitoring, with WHR adding value specifically for central adiposity risk stratification rather than general obesity classification.

### 4.2 The MVPA–BMI–PL mediation pathway

The mediation analysis confirmed that BMI partially mediated the association between MVPA and physical literacy. Each additional day of self-reported MVPA was associated with a lower BMI (Path a: β = −0.377, *p* < 0.001), while higher BMI was independently associated with lower PL scores after controlling for MVPA (Path b: β = −0.514, *p* < 0.001). This produced a significant indirect effect [β = 0.194, 95% CI (0.107, 0.291)], accounting for 8.5% of the total association. This pattern is theoretically coherent, as excess body weight may restrict children's ability to perform agility, endurance, and coordination tasks while also reducing willingness to engage in PA. Such limitations may hinder the accumulation of movement experiences required for the development of physical competence and confidence (42, 43). This is the first evidence of this pathway in a South Asian pediatric population, extending findings from Canadian and Spanish cohorts to a sociocultural context shaped by different economic and cultural influences on child development (38, 39). Sensitivity analysis using the full CAPL-2 composite score, including the Daily Behavior domain, produced a statistically consistent indirect effect [β = 0.335, 95% CI (0.192, 0.491)], representing 8.3% of the total association. This supports the robustness of the findings and suggests that the mediation effect was not driven by construct overlap between the MVPA predictor and the Daily Behavior component of the outcome measure.

The more notable finding was the magnitude of the direct effect. After accounting for the BMI-mediated pathway, MVPA remained strongly and positively associated with PL (*c*′ = 2.091, SE = 0.189, *p* < 0.001), representing approximately 91.5% of the total association. These findings suggest that PA supports PL largely through mechanisms independent of body weight. Repeated movement practice, mastery experiences, improved movement self-efficacy, and the motivational benefits of regular participation may all contribute to this relationship (44). Although the indirect effect through BMI was statistically significant, its relative contribution was modest compared with the dominant direct pathway. This distinction is important because it indicates that children may still experience substantial gains in PL even when increased PA does not immediately lead to noticeable reductions in body weight (45). A recent study among Chinese students across different obesity categories similarly reported that MVPA mediated the relationship between PL and physical fitness, while the direct effect of PL on fitness remained dominant regardless of obesity status (46). From a practical perspective, particularly in settings such as South Punjab where dietary modification and sustained weight reduction may be influenced by economic and cultural constraints (17–19), programmes that increase PA may substantially improve children's PL even when changes in body weight are limited. This reinforces the importance of promoting PA not only for weight management, but also for its broader contribution to movement competence, confidence, participation, and overall wellbeing (47).

Sex-stratified analyses showed that a slightly larger proportion of the MVPA–PL association was mediated through BMI among girls (10.2%) compared with boys (7.1%). This difference corresponded with a stronger Path b coefficient in girls (β = −0.552 vs. −0.475). The findings suggest that the physical and psychosocial burdens associated with higher BMI may have a greater influence on girls' physical literacy development, potentially because they interact with existing structural and cultural barriers that already limit girls' participation in physical activity within this context (30, 48). This sex-specific mediation pattern has not previously been reported in this population and should be examined further using longitudinal research designs capable of establishing the direction and stability of these associations over time.

A key limitation of the mediation analysis is the cross-sectional nature of the study, which prevents causal interpretation of the observed pathways. An alternative explanation, whereby higher PL promotes greater PA that subsequently contributes to lower BMI, is equally plausible and theoretically supported (14). The proposed model therefore reflects a theoretically informed developmental framework rather than an established causal sequence. Longitudinal research is required to determine temporal ordering and clarify the causal relationships among MVPA, BMI, and PL.

### 4.3 Sex differences and sociocultural context

A consistent finding across our analyses was that boys outperformed girls on every objective and self-reported physical activity indicator and on most physical literacy domains. Boys recorded an average of 8,115 ± 2,473 weekly steps compared with 7,611 ± 1,814 in girls, reported MVPA on 5.17 vs. 4.79 days per week, and attained higher composite PL scores (55.10 vs. 49.97 out of 100). These differences were not attributable to body size or developmental stage, since age, height, weight, and BMI did not differ significantly between sexes. The gap therefore reflects something other than physical maturation most plausibly, differential opportunity, social permission, and structured access to active play. This interpretation is supported by a small but consistent literature documenting the cultural, religious, and socioeconomic constraints on girls' physical activity participation in Pakistan. Laar et al. (49) have shown that female students in Pakistan face overlapping barriers to sports and PA participation, including restricted access to outdoor spaces, gender-segregated school facilities of unequal quality, parental concerns about modesty and safety, and limited public visibility of female role models in sport. These constraints accumulate from early childhood and shape the everyday choices through which PA habits, motor skills, and confidence in movement are built. The pattern observed in our data shows that girls are less active than boys despite comparable physical capacity.

The knowledge and understanding domain is notable in this context: boys and girls scored comparably, and K&U showed no significant correlation with MVPA or step count in either sex. Equal levels of declarative knowledge alongside unequal participation suggest that the gender gap is not rooted in a knowledge deficit girls are not less aware of PA guidelines than boys but in the structural conditions that determine whether that knowledge can be acted upon. This has direct implications for intervention design. Gender-equitable physical spaces, parental engagement around girls' activity, and school policies that normalize active participation for girls are likely to be more effective than educational content alone (50, 51). For girls with higher BMI, these structural barriers compound the physical and motivational constraints already identified in the mediation analysis, creating conditions in which multiple reinforcing disadvantages converge.

### 4.4 Relationship among the adiposity measures

BMI and waist circumference were meaningfully correlated in this sample (*r* = 0.354, *p* < 0.01), consistent with their shared sensitivity to overall body fatness. Hip circumference followed a similar pattern (*r* = 0.372 with BMI), while WHR diverged notably, showing a negligible and non-significant correlation with BMI (*r* = 0.048) alongside a moderate negative correlation with hip circumference (*r* = −0.454). This pattern confirms that WHR captures fat distribution rather than overall adiposity and should not be used as a proxy for general weight status in growing children, whose body proportions shift substantially across the 8–12-year age range (41). For population-level monitoring in this context, BMI remains the most practical and interpretively transparent primary measure, with waist circumference offering complementary information on central adiposity (52). The near-zero BMI–WHR correlation further cautions against treating these indices as interchangeable in epidemiological reporting, a distinction that has direct implications for how childhood adiposity data from South Punjab should be presented and compared internationally.

### 4.5 Status of physical activity, BMI, daily behavior, and physical literacy

Overall, 15.0% of children in this sample were classified as overweight (9.7%) or obese (5.3%), while a further 5.1% were underweight, a dual nutritional burden that reflects the complex public health landscape of contemporary South Punjab. The combined overweight and obesity prevalence observed here is lower than rates reported in earlier studies from Lahore (24.5%) (48), Hyderabad (18%) (19), and Multan (15%) (53), which may reflect methodological differences in classification criteria or genuine temporal variation across regions. Nonetheless, the prevalence of 15.0% OW/OB in school-aged children represents a substantial public health concern, particularly given that childhood obesity is associated with elevated long-term risk for type 2 diabetes, cardiovascular disease, and metabolic syndrome, and that these risks tend to track into adulthood when established early (54, 55).

In terms of daily physical activity, the largest share of children fell in the Progressing category for both step count (47.9%) and self-reported MVPA (47.7%), with only 14.9% and 14.7% reaching the Excelling level, respectively. The Daily Behavior domain showed a less favorable profile, with 56.4% of children classified as Beginning, indicating that the majority did not meet CAPL-2 norms for habitual daily activity. This pattern closely mirrors that of the composite PL score, where 55.7% of children were in the Progressing category and only 12.4% reached Excelling, while 15.9% remained at Beginning. Taken together, these distributions indicate that most children in South Punjab have not yet attained the levels of physical activity or physical literacy that CAPL-2 guidelines associate with adequate health and developmental outcomes for this age group.

Compared with children in Ontario, Canada, where substantially higher proportions meet the Achieving benchmark, the South Punjab sample shows a marked shortfall though this comparison should be interpreted cautiously given very different policy, infrastructure, and cultural contexts (56). Canada has longstanding national initiatives on PL which likely contribute to higher attainment. Comparisons with non-Western settings are more contextually appropriate. In Iran, Valadi and Cairney (57) found approximately 50% of children in the Progressing category for PL and DB, broadly comparable to our findings with fewer reaching the Excelling level. A CAPL-2 validation study in China similarly reported that only 16.6% of children achieved Achieving or Excelling in PL (58), compared with 28.4% in our sample, suggesting that children in South Punjab may be faring modestly better than some East Asian peers while still falling well short of Canadian benchmarks. These convergences across diverse non-Western settings suggest that the attainment gap relative to Canadian norms reflects a broader global pattern rather than a distinctly local problem.

The situation in South Punjab, where most of children have not yet reached recommended PA or PL levels, and where gender, weight status, and access to opportunity all intersect to compound disadvantages, calls for integrated efforts at the school and community level. Targeted physical education programmes, active school design, and community-based PA initiatives that are sensitive to local cultural norms around gender and physical participation represent the most promising levers for shifting these distributions over time.

### 4.6 Strengths, limitations, and future directions

This study has several important strengths that enhance the credibility and relevance of the findings. First, the study included a large and geographically stratified sample of school-aged children drawn from multiple divisions of South Punjab, providing broad regional representation within this underrepresented population. Second, the study utilized the culturally adapted and linguistically validated Urdu version of the CAPL-2 for Pakistani children, which strengthened the contextual appropriateness of the PL assessment. Another notable strength was the use of construct-independent correlation analyses to minimize part-whole inflation between physical activity indicators and PL outcomes. In addition, sex-stratified mediation analyses allowed a more detailed understanding of how the MVPA–BMI–PL relationship may differ between boys and girls. The sensitivity analyses, conducted using two different operationalization's of the PL outcome, also produced consistent findings, increasing confidence in the robustness of the observed mediation pathway.

Despite these strengths, several limitations should be acknowledged. Most importantly, the cross-sectional nature of the study limits interpretation to statistical associations and does not permit causal or temporal conclusions. Although the mediation analysis identified significant indirect pathways, these findings should not be interpreted as evidence that MVPA directly reduces BMI and subsequently improves PL. Reverse, and bidirectional relationships remain equally plausible. Self-reported MVPA may also be affected by recall bias and inaccuracies in children's perception of activity intensity, although the moderate agreement with objective pedometer-derived step counts provides some reassurance regarding the overall activity profile of the sample. Additionally, the study did not include potentially important confounding variables such as dietary intake, socioeconomic background, parental support, or psychosocial factors, all of which may influence children's PA participation, weight status, and PL development. Finally, because the sample was drawn exclusively from South Punjab, the findings may not fully generalize to other regions of Pakistan with different cultural, environmental, and socioeconomic conditions.

Several important directions for future research emerge from these findings. Longitudinal studies are needed to clarify the temporal relationships among MVPA, BMI, and PL across childhood and adolescence, and to determine whether the direct and indirect pathways identified in this study remain stable over time. Future work should also incorporate multilevel analytical designs capable of examining how school-level characteristics, including PE quality, access to facilities, teacher training, and recreational infrastructure, shape children's movement experiences and PL development. Perhaps most importantly, there is a clear need for gender-responsive intervention studies within South Punjab. Experimental research examining whether increased access to structured PA opportunities for girls leads to measurable improvements in PL would provide valuable practical evidence. Similarly, intervention programmes combining PA promotion with nutritional and behavioral support may help determine whether targeting both movement participation and weight-related barriers produces stronger outcomes than PA promotion alone. The present study provides an important regional baseline against which such future interventions and policy initiatives can be evaluated.

## 5 Conclusion

This study provides the first evidence from a Pakistani pediatric population that BMI plays a meaningful role in the association between MVPA and physical literacy. BMI statistically mediated part of the MVPA–PL relationship, suggesting that healthier weight status may support children's engagement in movement experiences linked with PL development. Children with overweight and obesity consistently demonstrated lower MVPA participation, lower daily step counts, and reduced PL scores compared with their normal-weight peers. Although the majority of the association between MVPA and PL remained direct, the findings highlight that weight-related factors should not be overlooked when examining children's PA and PL profiles in South Punjab.

Most children in this sample had not achieved recommended PL benchmarks, while girls consistently demonstrated lower PA and PL scores than boys despite comparable BMI and knowledge levels. These findings suggest that sociocultural and environmental barriers may influence participation opportunities, particularly for girls. Overall, the results emphasize the interconnected nature of PA, BMI, and PL during childhood and support the need for culturally relevant, gender-responsive, and school-based approaches that promote active participation while addressing weight-related barriers in under-resourced settings.

## Data Availability

The raw data supporting the conclusions of this article will be made available by the authors, without undue reservation.
